# Use of hospital palliative care according to the place of death and disease one year before death in 2013: a French national observational study

**DOI:** 10.1186/s12904-018-0327-z

**Published:** 2018-05-16

**Authors:** Claire Poulalhon, Laureen Rotelli-Bihet, Sébastien Moine, Anne Fagot-Campagna, Régis Aubry, Philippe Tuppin

**Affiliations:** 1 National sickness insurance fund, 26-50, avenue du Professeur André Lemierre, 75986 Paris cedex 20, France; 2 National Palliative care and end of life center, 75020 Paris, France; 3 Health Education and Practices Laboratory, Paris XIII University EA 3412, 93017 Bobigny cedex, France; 4 Pain and palliative Care Department, Teaching Hospital, 25030, Besançon, France

**Keywords:** Palliative care, Hospital, Death, Health status, Administrative health data, Place of death, Cancer, End-of-life care

## Abstract

**Background:**

Only limited data are available concerning the diseases managed before death and hospital palliative care (HPC) use according to place of death in France. We therefore conducted an observational study based on administrative health data in a large population to identify the diseases treated one year before death in 2013, the place of stay with or without hospital palliative care, and the place of death.

**Methods:**

French health insurance general scheme beneficiaries were identified in the National Health data Information System (Snds) with a selection of information. Diseases were identified by algorithms from reimbursement data recorded in the Snds database.

**Results:**

347,253 people were included in this study (61% of all people who died in France). Place of death was short stay hospital for 51%, Rehab (7%), hospital at home (3%), skilled nursing home (13%) and other (26%). Chronic diseases managed in 2013 before death were cardiovascular/neurovascular diseases (56%), cancers (42%), and neurological and degenerative diseases (25%). During the year before death, 84% of people were hospitalized at least once, and 29% had received HPC. HPC was used by 52% of cancer patients (lung cancer: 62%; prostate cancer: 41%). In the absence of cancer, the use of HPC varied according to the disease: acute stroke: 24%, heart failure: 17%, dementia: 17%, multiple sclerosis: 23%.

**Conclusions:**

Health administrative data can refine the knowledge of the care pathway prior to death and the HPC utilisation and can be useful to evaluate heath policies and improve monitoring and assessment of HPC use.

**Electronic supplementary material:**

The online version of this article (10.1186/s12904-018-0327-z) contains supplementary material, which is available to authorized users.

## Background

By improving survival, technical and scientific progress in the field of medicine has resulted in a growing burden of chronic diseases and has modified the temporality of the end of life, which may involve the use of palliative care earlier in the course of the disease [1]. In France, one in four people were over the age of 60 years in 2013 for a population of 65.6 million inhabitants, and people over the age of 60 will represent almost one-third of the population in 2060 [2]. This ageing population will lead to an increased number of people with chronic fatal diseases requiring palliative care [3]. Three types of end-of-life pathways have been distinguished: rapid decline, gradual decline with episodes of decompensation, and slow and progressive decline [4]. End of life care is therefore not confined to the last instants of life, but corresponds to a longer period with a high level of health care consumption designed to relieve psychological distress, the patient’s symptoms and provide support to caregivers.

In France, a new national plan for the development of end-of-life palliative care and supportive care was launched in 2015 [5]. The previous plan (2008–2012) revealed deficiencies concerning the possibility of maintaining people at home at the end of life with differences in terms of access according to age, place of residence and disease [6]. However, only limited data are available, for France and other countries, concerning the place of death, palliative care needs, palliative care use and end-of-life care pathways according to the patient’s characteristics and diseases. These data are derived from studies based on samples or subgroups determined according to the place of management or, more often, causes of death. Hospital diagnoses are less frequently studied using national healthcare administrative databases [7–19]. People needing palliative care in France have been estimated to represent 44% or 69% of deaths on the basis of causes of death associated with a theoretical indication for palliative care [7–9].

The objective of this observational study using administrative health data for a large population was to identify the diseases treated one year before death in 2013, the place of stay with and without hospital palliative care (HPC) and the place of death according to the characteristics of this population.

## Methods

### Population and settings

This observational study concerned all French national health insurance general scheme beneficiaries (77% of the French population) who died in 2013. The study was confined to this scheme because it systematically records the vital status of its beneficiaries. Individuals without at least one healthcare refund in 2012 and 2013, allowing identification of diseases, and children born in 2013, were excluded. The national health database (Snds) was used to study the various types of hospitals or institutions: short-stay hospitals with acute wards and palliative care facilities (specific wards, mobile teams providing palliative care advice and expertise to other healthcare professionals) and specific beds; rehabilitation (rehab) hospitals, to which people are usually admitted after an acute hospital stay and which are devoted to rehabilitation as well as palliative care depending on their rehabilitation specialization; and hospital at home (HaH) care delivered by hospital teams. Information on skilled nursing home (SNH) stays is available, but not for all retirement homes [20]. Information is not available for mobile teams not specifically attached to HaH units or for ambulatory palliative care provided by general practitioner. Palliative care is provided by specialists in hospital and mainly by general practitioners in SNH. Guidelines on specific pathways and best practices are published by the French National Authority for Health (Haute Autorité de santé, www.has-sante.fr) [21].

### Data sources

Data concerning the beneficiaries are collected in the Snds database, which comprises comprehensive, anonymous, individual data concerning all prescriptions, examinations and procedures reimbursed [22]. The Snds database does not contain information on clinical results related to visits, prescriptions or examinations. Nevertheless, it includes information on the presence of certain long-term diseases (LTD) eligible for 100% reimbursement of healthcare. All these data are linked to data concerning hospital stays provided by the French hospital discharge database (PMSI), and residence in SNH. LTD and hospital diagnoses are coded according to the international classification of diseases, 10th edition (ICD-10). There is no data on causes of death.

### Data and frequency analysis

HPC in short stay hospitals, Rehab and HaH was identified through palliative care diagnostic codes, specific palliative care beds or classification of the palliative care stay according to the various types of hospitalization. Use of HPC was calculated over the year preceding death including the date of death and at the date of death only. The totals indicated in the tables refer to people receiving HPC during the year before death or at the date of death during the last hospital stay. Information on place of death is available for deaths occurring during hospital stays (short stay hospitals, Rehab or HaH) or in a SNH. Nevertheless, among the “other places” of death, it is not possible to distinguish death at home and death outside home: in a retirement home, in public places, etc. The *Caisse Nationale d’Assurance Maladie* (Cnam) general health scheme fund has developed a tool based on Snds data with algorithms designed to identify beneficiaries reimbursed for chronic diseases and common, serious or expensive diseases and treatments, in order to study these diseases in terms of numbers, prevalence rates, expenditure and annual growth [23]. Information concerning the patients’ diseases in 2012 and in 2013 was derived from this tool (Additional file 1). Algorithms identify 56 non-exclusive groups of diseases, classified into 13 main categories, based on principal diagnoses, related or significant associated diagnoses in short-stay hospitals and psychiatric hospitals coded according to ICD-10; long-term disease diagnoses (LTD); dispensing of specific drugs; and specific procedures. Schematically, the term “acute” refers to hospitalization during the year with an appropriate ICD-10 code and the chronic phase is defined by attribution of LTD status during the year and/or hospitalization with specific codes during the last 5 years. The acute episode always takes precedence to a chronic phase and these two groups are mutually exclusive for a given disease. Cancer is defined by short-stay hospitalizations over a 5-year period (specific cancer diagnoses and chemotherapy and radiotherapy codes) and/or LTD status attributed over the previous 2 years. In Tables 2 and 3, two groups were selected: patients with at less a diagnosis of a cancer and patients without diagnosis of cancer but with one or more other diseases. These definitions were submitted to sensitivity analyses concerning the numbers of subjects and the expenditure incurred, and a critical review was conducted by an independent partner, allowing improvement of these algorithms [24].

**Fig. 1:**
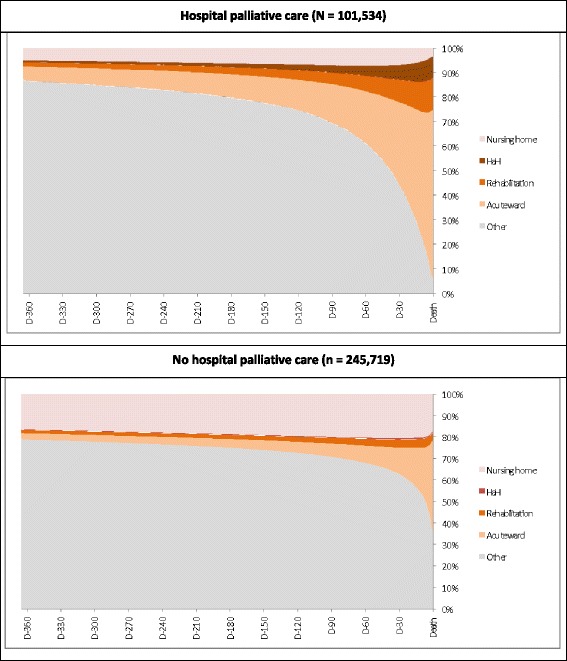
Setting on a given day the year preceding death and hospital palliative care use

Characteristics of people died in 2013 were described including age, gender, residence in a SNH, diseases identified by algorithms, and the presence of at least one admission the year before death in each type of hospital and/or on the day of death. Rates of subjects with HPC among all the subjects died in each type of hospital were also calculated according to subject characteristics and diseases. Figure 1 represents the place of stay of beneficiaries on a given day during the year preceding death.

All frequencies were calculated with SAS Enterprise Guide software (version 4.3; SAS Institute Inc., Cary, NC, USA).

## Results

### Characteristics

A total of 347,253 general scheme beneficiaries who died in 2013 were included (49.7% men and 50.3% of women) (Table 1). The mean age of death was 77 years (73 years for men, 81 years for women). Deaths before the age of 85 years predominantly concerned men, with a particularly marked sex difference between the ages of 15 and 35 years.

**Table 1:** Diseases managed of general scheme beneficiaries who died in 2013, according to sex and age

	Total	Sex		Age								
		Men	Women	< 15	15–34	35–44	45–54	55–64	65–74	75–84	85–94	≥ 95
N	347,253	172,591	174,662	1066	4409	7030	18,860	39,656	50,638	89,933	114,652	21,009
%	100.0	49.7	50.3	0.3	1.3	2.0	5.4	11.4	14.6	25.9	33.0	6.1
	%	%	%	%	%	%	%	%	%	%	%	%
Women	50.3	–	–	42.6	32.3	37.2	37.2	34.0	35.6	46.7	63.4	80.9
Age (mean years)	77.0	73.2	80.7	–	–	–	–	–	–	–	–	–
Skilled nursing home resident	21.1	12.8	29.3	–	–	–	–	1.8	5.1	14.0	30.4	42.5
At least one hospitalisation during the year	83.5	85.4	81.6	81.1	59.1	70.6	80.2	85.5	88.5	87.6	82.8	66.5
Short stay hospitals	82.8	84.9	80.7	80.9	58.8	70.1	79.7	85.1	88.1	86.9	81.9	65.3
Rehabilitation	22.6	21.3	24.0	8.4	5.8	8.9	12.9	16.0	19.9	26.4	27.2	17.8
Hospital at home	6.6	7.0	6.2	12.7	6.1	8.2	9.5	10.1	9.3	6.8	4.1	2.6
Diseases managed												
Cardiovascular/neurovascular diseases	56.5	58.4	54.6	16.5	11.4	16.3	24.6	37.9	50.8	63.2	68.8	62.4
Coronary heart disease	20.5	24.6	16.4	0.5	1.2	3.6	7.8	13.9	20.4	24.3	24.0	20.3
Acute stroke	4.9	4.2	5.7	1.3	1.6	2.9	2.9	3.1	4.0	5.9	6.0	3.8
Stroke sequelae	9.5	9.6	9.4	5.1	2.7	2.8	4.0	5.9	8.4	10.8	11.7	10.4
Heart failure	24.3	23.0	25.7	5.3	3.5	4.5	6.5	10.5	17.2	26.7	34.2	32.0
Peripheral artery disease	10.7	13.8	7.6	0.5	0.5	1.1	4.1	9.7	12.5	12.6	11.2	9.0
Arrhythmia	27.5	28.0	27.0	4.0	3.1	4.6	6.9	12.3	21.1	32.5	37.4	28.0
Valvular disease	6.8	6.5	7.0	0.3	0.8	1.5	1.7	3.0	5.4	8.5	9.0	5.4
Pulmonary embolism	1.1	1.0	1.2	0.2	0.5	0.9	1.1	1.4	1.5	1.2	1.0	0.5
Diabetes	21.1	23.5	18.7	0.2	2.1	5.3	10.6	19.5	28.5	27.6	18.9	10.3
Cancers	42.1	48.7	35.6	20.6	17.5	31.2	47.1	58.3	58.7	46.6	31.1	17.9
Female breast	4.8	–	9.5	0.0	1.6	5.8	6.7	5.7	5.5	4.9	4.0	3.1
Colon	5.8	6.4	5.2	0.2	1.0	3.0	4.1	6.0	7.5	7.3	5.0	3.1
Lung	6.5	9.5	3.5	0.0	0.9	5.0	11.5	15.7	12.6	5.8	1.8	0.4
Prostate	5.1	10.2	–	0.1	0.0	0.2	0.6	2.2	5.7	7.6	5.6	2.2
Other cancers	26.3	30.4	22.3	20.6	14.9	20.3	29.2	36.2	37.1	29.4	19.0	10.7
Psychiatric disorders	9.7	9.7	9.8	4.8	21.7	27.1	22.6	15.2	10.0	8.0	6.5	4.4
Psychotic disorders	1.8	1.9	1.8	0.1	6.7	7.3	5.1	3.2	2.2	1.4	0.8	0.5
Manic disorders	0.8	0.65	1.0	0.0	1.5	2.5	1.7	1.4	1.0	0.8	0.4	0.2
Depression	2.9	2.3	3.4	0.1	4.8	7.1	6.1	4.1	2.7	2.5	2.2	1.6
Addictive disorders	2.0	3.0	0.9	0.0	8.2	12.2	10.1	5.1	1.9	0.6	0.2	0.1
Psychotropic treatments^a^	27.2	23.9	30.5	4.4	10.1	16.0	20.3	22.9	24.9	27.9	31.2	30.6
Neurological or degenerative diseases	24.6	19.7	29.4	18.6	10.5	10.4	9.7	8.8	11.8	25.7	37.1	33.1
Dementias (Alzheimer)	18.7	13.3	24.1	0.2	0.3	0.4	0.8	1.8	5.6	19.6	32.6	30.3
Parkinson’s disease	3.9	4.1	3.7	0.1	0.1	0.0	0.2	0.7	2.4	5.8	5.3	2.8
Multiple sclerosis	0.2	0.2	0.3	0.0	0.3	0.6	0.6	0.6	0.4	0.2	0.1	0.0
Paraplegia	0.4	0.5	0.3	1.2	1.3	1.1	0.7	0.6	0.5	0.3	0.2	0.1
Myopathy, myasthenia	0.2	0.3	0.2	2.5	1.3	0.6	0.5	0.4	0.3	0.2	0.1	0.1
Epilepsy	2.8	3.0	2.6	11.5	5.6	6.6	5.9	4.1	2.9	2.4	2.0	1.2
Chronic respiratory diseases	20.5	24.2	16.8	19.0	7.0	10.4	16.0	22.6	25.1	24.0	18.3	12.3
Inflammatory or rare diseases or HIV or AIDS	4.3	3.9	4.6	9.4	3.9	5.0	5.4	4.4	4.6	4.8	3.9	2.0
Treated renal failure	1.4	1.8	1.1	0.1	0.6	1.1	1.3	1.5	2.4	2.0	0.9	0.2
Diseases of the liver or of pancreas	8.9	11.5	6.4	9.2	7.7	15.0	20.6	17.9	14.7	7.9	3.2	1.3
Other long-term diseases	10.8	9.0	12.6	15.6	7.1	6.6	6.8	7.9	9.0	10.6	12.9	15.6
Maternity	0.05	–	0.1	–	1.6	1.1	0.1	–	–	–	–	–
None of health states above	4.4	4.9	3.9	34.4	38.2	19.2	10.0	5.1	2.7	2.1	2.8	6.7

^a^with or without psychiatric disorders

Globally, the diseases most commonly managed before death were cardiovascular/neurovascular diseases (56%), especially heart failure (24%), particularly in women and the oldest people, and coronary heart disease (20%), which was more frequent in men. Deceased people had been managed for cancer in 42% of cases (close to 60% between the ages of 55 and 74 years, men: 49%; women: 36%), dementia in 19% of cases (men: 13%; women: 24%), and chronic respiratory disease in 20% of cases (men: 24%: women: 18%).

### Hospitalizations and place death

During the year preceding death, 83% of people were hospitalized at least once (short stay hospitals: 83%, Rehab: 23%, HaH: 7%) (Table 1). These proportions were slightly higher for men except for hospitalizations in rehabilitation units (men: 21%; women: 24%). Analysis according to age revealed a high hospitalization rate before the age of 15 years (81%), lower rates between the ages of 15 and 34 years (59%), followed by rates that gradually increased with age until the 75 to 84 years age-group (88%). Although the frequency of at least one Rehab admission to a rehabilitation unit increased regularly with age, the frequency of HaH decreased from the 65–74 years age-group. Fig. I present a dynamic view of the proportion of people in each place of residence on a given day during the year before death. The proportion of people receiving HPC at least once during the year and admitted to a short stay hospital on a given day rapidly increased over the months preceding death: 9% six months before death, 16% three months before death, 34% one month before death and 69% on the day of death. An increased admission rate was also observed for rehabilitation units (one month before death: 9%, on the day of death: 13%) and HaH (6% vs 9%). Inversely, the proportion of people not hospitalized and not living in a SNH decreased markedly (one month before death: 44%, 14 days before death: 29%, on the day of death: 6%). The proportion of people living in a SNH started to decrease three weeks before death (6%) to reach 3% on the day of death. These variations differed or were less marked in the absence of HPC during the year before death, with a moderate increase for short stay hospital admissions (six months before death: 4%, three months before death: 6%, one month before death: 13%, on the day of death: 44%), stable Rehab admissions (4%), and a moderate reduction for “other places” of residence (one month before death: 63%, 14 days before death: 55%, on the day of death: 35%) and SNH (three weeks before death: 21%, on the day of death: 17%).

Overall, death occurred in hospital for 58% of deceased people (51% in short stay hospitals, 7% in Rehab), in HaH for 3%, in SNH for 13% and in “other places” for 26% (public place, home, retirement home, etc.) (Table 2). A higher proportion of women died in SNH (19% vs 7% for men), but with a different mean age. A higher proportion of men died in short stay hospitals (55% vs 47% for women) and in “other places” (29% vs 24%). The proportion of deaths in SNH increased markedly after the age of 85 years, while the proportion of deaths in “other places” reached a peak of 60% between the ages of 15 and 34 years and the proportion of deaths in short stay hospitals reached a peak of 60% between the ages of 55 and 74 years. The proportion of deaths in rehabilitation units increased slightly with age and the proportion of deaths in HaH decreased after the age of 74 years. Sixty-four per cent of people managed for cancer died in a short stay hospital, 7% died in a SNH and 16% died outside of a hospital, HaH or a SNH. The proportions of deaths in SNH (16%) and outside of a hospital, HaH or a SNH (23%) were higher among people with cardiovascular diseases (other than cancer) than for the overall population. These proportions were even higher for people with neurological or degenerative diseases: death in a NSH: 35% and death outside of a hospital or a NSH: 23%.

**Table 2:** Place of death in 2013 according to the general scheme beneficiaries characteristics and diseases managed

Place of death		Short stay hospital	Rehab	Hospital at home	Skilled nursing home	Other
	n^a^	%	%	%	%	%
Total	347.3	51.0	6.5	2.8	13.3	26.4
Men	172.6	54.8	6.1	2.9	7.3	28.9
Women	174.7	47.1	6.8	2.8	19.3	23.9
Age (years)						
< 15	1.1	59.7	1.4	4.2		34.7
15–34	44	37.6	0.9	1.7		59.7
35–44	70	45.8	1.6	2.3	0.1	50.2
45–54	189	55.4	3.0	2.8	0.2	38.6
55–64	397	60.7	4.5	3.5	1.2	30.2
65–74	506	62.0	5.6	3.6	3.2	25.7
75–84	89.9	55.3	7.7	3.1	9.8	24.1
85–94	1147	43.6	8.0	2.3	23.5	22.6
≥ 95	210	27.5	5.2	1.8	40.2	25.3
Cancers	146.2	63.6	8.3	4.7	7.0	16.4
Female breast	16.5	58.9	8.2	4.5	11.1	17.3
Colon	20.1	62.0	8.9	5.0	8.0	16.1
Lung	22.5	71.1	8.2	5.0	2.3	13.4
Prostate	17.6	56.5	8.3	4.4	8.9	21.9
Other cancers	91.4	64.9	8.4	4.9	6.5	15.4
Other diseases in the absence of cancer						
Cardiovascular/neurovascular diseases	121.4	53.2	6.5	1.6	16.1	22.6
Coronary heart disease	45.0	56.1	6.3	1.4	13.0	23.2
Acute stroke	12.9	75.5	7.9	1.3	9.1	6.1
Stroke sequelae	21.7	46.2	6.3	2.1	21.9	23.4
Heart failure	57.3	59.3	7.1	1.6	13.9	18.1
Diabetes	43.3	48.2	5.7	1.7	12.5	31.9
Psychiatric disorders	23.9	34.8	4.0	0.8	16.2	44.1
Neurological or degenerative diseases	63.4	37.4	6.0	2.3	31.7	22.6
Dementias (including Alzheimer)	50.2	35.4	6.4	2.2	35.8	20.2
Parkinson’s disease	10.0	38.9	6.1	2.6	28.0	24.4
Multiple sclerosis	0.6	48.5	1.8	5.0	12.0	32.7
Paraplegia	0.8	51.0	5.1	3.8	10.1	30.0
Myopathy or myasthenia	0.6	58.4	3.1	3.1	5.9	29.4
Epilepsy	6.1	44.5	4.5	2.3	18.0	30.8
Chronic respiratory diseases	39.0	56.2	5.7	1.6	10.4	26.1
Inflammatory or rare diseases, HIV or AIDS	9.0	55.2	6.1	1.7	11.8	25.2
Treated renal failure	3.3	72.2	3.1	1.6	5.1	18.0
Diseases of the liver or pancreas	14.6	70.5	4.5	1.0	4.2	19.9
Other long-term diseases	24.2	42.9	5.6	2.0	19.8	29.7

^a^Thousand

### Use of hospital palliative care

Twenty-nine per cent of all deceased people received hospital palliative care at the date of death or during the year preceding death (Table 3). This proportion varied little according to sex (men: 30%; women: 28%), but more markedly according to age, with 30% for people under the age of 15 years and a peak around 39% between the ages of 55 and 74 years; and also according to CMU-C status (presence: 28%; absence: 34%). People managed for cancer received HPC in 52% of cases (lung cancer 62%; prostate cancer: 41%), while people with other diseases, excluding cancer, received HPC in 24% of cases for acute stroke, 17% of cases for heart failure, 23% of cases for multiple sclerosis, 18% of cases for Parkinson’s disease, 17% of cases for dementia, 16% of cases for chronic respiratory disease and 20% of cases for treated renal failure. In more than one-half of cases, HPC was only provided during the stay associated with death (13% of people received HPC before the stay resulting in death, while 16% only received HPC during the stay resulting in death, for a total of 29% of people receiving HPC). Access to HPC during the year before death decreased with age and was less frequent in the absence of cancer. Among the people who died in a NSH, not equipped with a palliative care unit identified in the Snds database, 8% had previously received HPC, especially between the ages of 65 and 84 years and in the presence of a cancer (17%). HPC before death was also observed in the “Other places” category (7%), with higher rates for older people, in the presence of a cancer (19%) and, to a lesser extent, in the presence of multiple sclerosis (9%) or acute stroke (11%). Thirty-five per cent of people who died during other types of hospitalization than short stay hospitals (in rehabilitation units or HaH) had received HPC before death and 69% had received HPC at the date of death.

**Table 3:** Hospital palliative care use at the time of death, during the last year (total), or before death for people died in a skilled nursing home or in another place according to sex, age and disease managed

Place of deaths	All places			Short stay hospital		Rehab		Hospital at home		Skilled nursing home#		Other^b^	
	N^a^	Death°	Total	N^a^	Total	N^a^	Total	N^a^	Total	N^a^	Before death	N^a^	Before death
		%	%		%		%				%		%
Total	347.3	15.7	29.2	176.9	39.3	22.51	60.0	9.82	88.9	46.3	7.6	91.7	6.7
Men	172.6	16.6	30.9	94.6	40.1	10.55	60.9	4.97	90.2	12.6	9.6	49.8	6.5
Women	174.7	14.9	27.6	82.3	38.5	11.96	59.1	4.84	87.5	33.8	6.8	41.8	7.0
Age (years)													
< 15	1.1	11.5	30.3	0.6	38.2	0.02	93.3	0.05	95.6		–	0.4	6.2
15–34	4.4	6.6	15.8	1.7	33.3	0.04	58.5	0.08	100		–	2.6	1.6
35–44	7.0	10.7	25.4	3.2	45.4	0.11	68.4	0.16	95	0.0	–	3.5	2.6
45–54	18.9	15.9	34.5	10.4	49.7	0.56	80.0	0.54	96.6	0.0	–	7.3	4.8
55–64	39.7	19.4	39.9	24.1	50.5	1.77	79.4	1.38	95.3	0.5	16.0	12.0	7.3
65–74	50.6	19.8	38.8	31.4	46.5	2.81	71.1	1.81	92.8	1.6	14.0	13.0	8.5
75–84	89.9	17.6	31.7	49.8	38.2	6.92	63.3	2.78	89.1	8.8	10.0	21.6	8.2
85–94	114.7	13.3	22.2	50.0	29.9	9.20	50.9	2.66	81.7	26.9	7.3	25.9	6.6
≥ 95	21.0	8.4	13.2	5.8	25.6	1.09	42.9	0.37	77.8	8.4	3.8	5.3	3.9
Cancers	146.2	25.9	51.9	93.0	57.8	12.19	76.4	6.86	94.5	10.3	18.0	23.9	19.0
Female breast	16.5	24.5	50.1	9.7	59.6	1.35	75.7	0.74	93.9	1.8	15.0	2.9	17.0
Colon	20.1	25.9	52.7	12.5	57.9	1.80	76.8	1.01	94.6	1.6	20.0	3.2	22.0
Lung	22.5	30.5	62.5	16.0	65.5	1.84	84.3	1.12	97	0.5	35.0	3.0	25.0
Prostate	17.6	20.8	40.9	9.9	48.0	1.46	67.2	0.78	92.2	1.6	14.0	3.8	13.0
Other cancers	91.4	26.4	53.3	59.3	58.5	7.68	77.1	4.47	94.7	5.9	19.0	14.0	20.0
Other diseases in the absence of cancer													
Cardiovascular/neurovascular diseases	121.4	10.7	15.9	64.6	19.0	7.88	40.3	1.96	74.9	19.5	6.4	27.5	4.0
Coronary heart disease	45.0	*10.0*	14.9	25.3	17.4	2.82	38.3	0.63	76.5	5.9	6.3	10.4	3.6
Acute stroke	12.9	*18.2*	24.3	9.7	22.6	1.02	51.6	0.17	77.9	1.2	16.0	0.8	11.0
Stroke sequelae	21.7	*11.5*	18.0	10.0	24.2	1.37	41.5	0.46	76.6	4.8	6.0	5.1	5.3
Heart failure	57.3	*11.5*	17.3	34.0	19.1	4.07	39.3	0.90	78	8.0	7.0	10.4	5.2
Diabetes	43.3	9.2	14.1	20.9	18.3	2.45	40.2	0.76	75.8	5.4	6.1	13.8	2.9
Psychiatric disorders	23.9	6.6	10.5	8.3	19.1	0.96	36.9	0.20	76.2	3.9	5.1	10.6	1.9
Neurological or degenerative diseases	63.4	10.9	17.4	23.7	27.1	3.83	44.9	1.44	75.4	20.1	5.3	14.3	5.0
Dementias (including Alzheimer)	50.2	*11.0*	17.2	0.0	27.4	3.21	44.7	1.10	75	0.0	5.4	0.0	5.4
Parkinson’s disease	10.0	*11.6*	17.7	17.8	27.4	0.61	46.8	0.26	71.7	18.0	4.6	10.1	4.0
Multiple sclerosis	0.6	*10.2*	23.2	3.9	28.9	0.01	63.6	0.03	80	2.8	8.3	2.4	9.2
Paraplegia	0.8	*10.4*	20.6	0.3	26.2	0.04	44.2	0.03	62.5	0.1	5.9	0.2	6.7
Myopathy or myasthenia	0.6	*14.2*	21.2	0.4	27.4	0.02	55.6	0.02	72.2	0.1	2.9	0.3	3.6
Epilepsy	6.1	*11.3*	18.4	0.3	26.3	0.27	52.8	0.14	75.5	0.0	7.2	0.2	4.4
Chronic respiratory diseases	39.0	10.4	16.5	2.7	19.9	2.24	37.7	0.61	77.4	1.1	7.1	1.9	4.4
Inflammatory or rare diseases, HIV or AIDS	9.0	11.0	17.3	21.9	20.9	0.55	44.2	0.15	81.3	4.1	6.3	10.2	3.8
Treated renal failure	3.3	12.4	20.4	4.9	22.2	0.10	46.0	0.05	80.8	1.1	14.0	2.3	5.3
Diseases of the liver or pancreas	14.6	10.7	16.7	2.3	17.5	0.65	47.2	0.15	86.4	0.2	11.0	0.6	4.7
Other long-term diseases	24.2	9.7	15.1	10.3	21.5	1.36	40.0	0.48	74.5	0.6	5.3	2.9	3.7

^a^Thousand

° Death: hospitalization in which death occurred; Total: hospital palliative care during the last year including hospitalization in which death occurred

^b^ No hospital palliative care identifiable in the database at the date of death

## Discussion

In a large population of deceased people, 60% died in a hospital setting and one-half died in a short stay hospital. The proportion of people hospitalized in a short stay hospital increased rapidly over the three months prior to death. The main chronic diseases managed one year before death were cardiovascular/neurovascular diseases (56%), cancers (42%), and neurological and degenerative diseases (25%). During the year before death, 84% of people were hospitalized at least once, and 29% received HPC. HPC was more frequently used by cancer patients, and, in the absence of cancer, by patients with stroke: 24%, heart failure: 17%, dementia: 17%, multiple sclerosis: 23%.

### Comparisons of places of death and diagnosis with French mortality statistics

This study was based on very comprehensive administrative and hospital reimbursement data and a large national study population [22]. The results can be compared with the national population and mortality statistics. Characteristics of deceased general scheme beneficiaries are relatively similar to those of all people who died in France in 2013, but with a smaller proportion of older people compared to the overall population (mean age: men: 73 years vs 79 years, women: 81 vs 85 years) [25]. General scheme beneficiaries are globally younger than beneficiaries of other schemes, which explain why this study included only 61% of all deaths in France, for 77% of the population.

In all deaths in 2013 in France, 57% occurred in hospital, 12% in retirement homes, 25% at home and 6% in a public place or in “Other places” [25]. These figures are similar to those reported in this study: 58% of people died in hospital, 13% died in a SNH and 29% died in “other places” (including 3% in HaH). However, SNH do not include all retirement homes and the proportion of SNH is unknown. This study was unable to directly estimate the proportion of deaths at home, but 29% of deaths occurred outside of hospitals and SNH and therefore theoretically at home (including HaH) or in a public place. As reported in other countries, a very great majority of French people (85%), free of any serious diseases, prefer to die at home [26]. However, in this study, only 42% of people died at home if SNH and “Other places” were considered to be home. This proportion decreased to 36% after subtracting the 6% of nationwide deaths in a public place [25].

End of life can be associated with a context of multiple diseases that cannot be reflected by a single leading cause of death. The distribution of diagnoses identified by subject in our study does not correspond to all causes of death in 2013: tumours (42% in this study for 29% as the leading cause of death), cardiovascular disease (56% for 25%), respiratory tract disease (20% for 7%), and dementia (19% for 4%) [25]. However, some causes of death were not included in the diagnoses because they could not be identified using the Snds database algorithms.

### Hospital palliative care use

Twenty-nine per cent of all deceased people received HPC at the date of death or during the year preceding death (16% at the date of death only, and 13% at least before death). These rates were 39% for deaths in short stay hospitals and 44% for all deaths in health care institutions. These rates correspond to a first estimate of HPC use in a large population in France and few data on this subject using hospital diagnosis are available in the international literature (15–17, 19). The results of published studies correspond more to HPC needs, than HPC use, estimated by algorithms using causes of death likely to benefit from palliative care. HPC needs vary according to the causes of death eliminated or included in the algorithm and their specific distributions in different populations [8]. Hospital diagnoses matched with causes of death were also used in an Australian study [19]. The number of people with palliative care needs in France in 2008 was estimated to be 41% of all deaths according to the method of Rosenwax et al. and 69% according to the method of Murtagh et al., which includes more causes of death [8, 27], such as chronic cardiovascular / neurovascular diseases other than heart failure, non-Alzheimer’s dementia and respiratory diseases other than chronic obstructive pulmonary disease. Thus, considering available data, estimation of palliative care need by health policymakers could be over or underestimated when national institution. Hospital diagnosis and multiple cause-of-deaths must be considered because cancer diagnosis may not be notified as the main or primary cause of death. While it is recommended to initiate palliative care earlier before death, the rate of palliative care use before death is relatively low in this study, except in rehabilitation units and HaH, which can constitute a short-stay alternative providing more specialized services in this field.

The frequency of palliative care before and at the date of death varies according to the type of disease, as well as their age and sex distributions, such as the classical high proportion of external causes in young boys. The type of palliative care can also vary according to age and type of disease. Analysis of HPC use by disease therefore appears to be more relevant than by causes of death, although other characteristics can also influence management, such as gender, age, and comorbidities.

### Strength and limits

Use of palliative care in France is probably underestimated because this study does not take into palliative care managed by the general practitioner at home or palliative care provided in NSH, as no specific markers, such as drug reimbursements, are available due to the lack of specificity. However, 5% of deceased people received HPC before death in a NSH or in “other places”, suggesting return or continued management of the patient at home or in a NSH, but the intensity of this care cannot be assessed.

The diseases managed can be identified by multisource algorithms such as those used in this study. These algorithms have been submitted to sensitivity analyses and expert reviews [24]. However, they depend on the use, offer and access to care. The data analyzed in this study were derived from administrative databases with their classical limitations concerning their primary objective, i.e. data collection and coding.

As part of the process of reflection on the end of life, the Snds can be used to describe end-of-life pathways and places of death and will soon be linked to causes of death. In the context of the new national palliative care plan in France, the Snds could be used to monitor several indicators focusing on HPC use, place of death but also to conduct more specific analyses in order to improve or evaluate specific subgroups (people with cancers, younger or older people, treatments and drugs used before death according to the disease).

## Conclusion

This study suggests a relatively high level of HPC use in France, especially for certain diseases and in HaH and rehab hospitals. Few people were hospitalized with HPC care recorded in the database before the last hospitalization before death or before return home. In the context of a reflection on the end of life, these results must be refined in order to elucidate various specific aspects (diseases, disparity of use, etc.) based on Snds data, but also with the help of healthcare professionals and patients, in order to guide end-of-life health policies and evaluate heath policies and improve monitoring, such as the national plan, and assessment of HPC use.

## Additional file

Additional file 1: Algorithms designed to identify beneficiaries reimbursed for chronic diseases and common, serious or expensive diseases and treatments. (DOC 102 kb)

## Abbreviations

Cnam: *Caisse Nationale d’Assurance Maladie* general health scheme fund
HaH: Hospital at home
HPC: Hospital palliative care
ICD-10: International classification of diseases, 10th edition
LTD: Long-term diseases
PMSI: Programme de médicalisation des systèmes d’information (PMSI) [Medical Information System Programme]
Rehab: Rehabilitation unit
SNH: skilled nursing homes; Snds Système National des données de santé [National Health data Information System]
